# The role of evaluation in iterative learning and implementation of quality of care interventions

**DOI:** 10.1080/16549716.2021.1882182

**Published:** 2021-06-20

**Authors:** Nikhil Shah, Sharon Mathew, Amanda Pereira, April Nakaima, Sanjeev Sridharan

**Affiliations:** a The Evaluation Centre for Complex Health Interventions, Dalla Lana School of Public Health, University of Toronto, Toronto, ON, Canada; b Bill and Melinda Gates Foundation, Munirka, Delhi, India

**Keywords:** Evaluation, quality of care, iterative learning, person-centred care

## Abstract

**Background**: The Lancet Global Health Commission (LGHC) has argued that quality of care (QoC) is an emergent property that requires an iterative process to learn and implement. Such iterations are required given that health systems are complex adaptive systems.

**Objective**: This paper explores the multiple roles that evaluations need to play in order to help with iterative learning and implementation. We argue evaluation needs to shift from a summative focus toward an approach that promotes learning in complex systems. A framework is presented to help guide the iterative learning, and includes the dimensions of clinical care, person-centered care, continuum of care, and ‘more than medicine. Multiple roles of evaluation corresponding to each of the dimensions are discussed.

**Methods**: This paper is informed by reviews of the literature on QoC and the roles of evaluation in complex systems. The proposed framework synthesizes the multiple views of QoC. The recommendations of the roles of evaluation are informed both by review and experience in evaluating multiple QoC initiatives.

**Results**: The specific roles of different evaluation approaches, including summative, realist, developmental, and participatory, are identified in relationship to the dimensions in our proposed framework. In order to achieve the potential of LGHC, there is a need to discuss how different evaluation approaches can be combined in a coherent way to promote iterative learning and implementation of QoC initiatives.

**Conclusion**: One of the implications of the QoC framework discussed in the paper is that time needs to be spent upfront in recognizing areas in which knowledge of a specific intervention is not complete at the outset. This, of course, implies taking stock of areas of incompleteness in knowledge of context, theory of change, support structures needed in order for the program to succeed in specific settings. The role of evaluation should not be limited to only providing an external assessment, but an important goal in building evaluation capacity should be to promote adaptive management among planners and practitioners. Such iterative learning and adaptive management are needed to achieve the goals of sustainable development goals.

## Background

### Evaluations of quality of care in a sustainable development goal (SDG) era

In its recent publication, The Lancet Global Health Commission (LGHC) on ‘High-Quality Health Systems in the SDG Era’ [1] poses the following provocative question: ‘What should a high-quality health system look like in countries with resource constraints and competing health priorities that aspire to reach the SDGs?’ The LGHC’s response to this question is informed by a recognition that health systems are complex adaptive systems and that such systems can resist change and can be impervious to isolated interventions [2]. Further, the LGHC argues that ‘quality of care is an emergent property that requires shared aims among all health system actors, favorable health system foundations, and is honed through iterative efforts to improve and learn from successes and failures’ [1].

Throughout this paper, the question we focus on is: What should evaluation’s role be in such iterative learning? The notion of quality of care (QoC) as an emerging aspect in complex adaptive systems demands a more comprehensive discussion around the role of evaluations in helping systems move toward greater quality in an iterative way. Such thinking about quality is especially critical in resource-constrained settings and where ideas of best practices might be difficult to implement given the constraints and complexities of specific settings [3,4].

Given the varieties of quality of care (QoC) interventions, this paper is not intended to provide a ‘how to’ do evaluation. We work in the field of evaluating complex interventions and our view is that the ‘how to’ do evaluations might depend on both the needs of stakeholders and the complexities of the interventions. Instead, our goal here is to raise awareness of the types of questions and alternative evaluation approaches can help with in planning, translating knowledge, and estimating impacts of QoC interventions. There are multiple purposes of evaluation; there is a need for the field of QoC program planners and implementers to be aware of these multiple evaluation approaches. We believe becoming aware of the wide variety of evaluation purpose and approaches available will help improve the quality of evaluation of QoC interventions [5].

How can a scientific approach to evaluation help in moving toward such an iterative approach? In recent years, the field of evaluation [3,6,7] has undergone a shift in thinking from a purely summative exercise (‘does an intervention work?’) toward a recognition that evaluations in complex systems [7–9] require a focus on helping an intervention or a system learn to develop more completely. Developmental evaluation [10,11], for example, accepts that evidence-based interventions typically need further development to adapt to specific contexts – the evaluation itself is helpful in aiding such development. Similarly, realist evaluators [12,13] explore the specific contexts and mechanisms that are necessary for interventions to work.

Recent discussions about QoC have increasingly revolved around the question of incorporating a multi-dimensional view of quality. The literature has also seen a growth in scholarly debate around related concepts of dignity and equity [14]; person-centered care [15–17]; and rights-based approaches [18]. Returning to an emergent view of QoC, the current paper focuses on how evaluations can help come to terms with such a multi-dimensional view of QoC that is grounded in the need for iterative learning. In our view, this means moving away from a narrow ‘what works’ evaluation to a role of evaluation that is sensitive to the development of QoC over time.

Building an iterative-learning system needs to go beyond a series of commissioned evaluations done by external organizations; instead, the focus needs to be how different parts of systems at various levels – national, state, district, regional, community, and facility can learn about ongoing improvement. There is a need to move from debates about best methods toward a clearer view of pathways of system-level learning.

### Outline of paper

This paper discusses what it means, from an evaluation perspective, to think about quality as an emergent, multi-dimensional construct that requires an iterative process to learn and implement [3]. To facilitate this, we propose a framework for QoC and focus our discussion on maternal health. While several QoC frameworks exist in the literature, not all of these frameworks provide a blueprint for approaching QoC as a multi-dimensional construct. The LGHC has argued that the sheer breadth of quality deficits in maternal and neonatal care means incremental solutions will be insufficient; the framework presented in this paper can help position the evaluations to create a more robust iterative process to achieve high quality, rather than exist as a single incremental change as they often do.

### Quality of care in maternal health

The need for an iterative evaluative approach to improving QoC becomes important when one considers the successes and failures of the millennium development goals (MDGs). The fourth and fifth MDGs were the global reduction of child mortality and the improvement of maternal health [19]. Despite the significant progress made toward these goals, every year more than 35,0000 women die due to complications related to pregnancy and childbirth [20]. Significant resources have been allocated to interventions designed to lower maternal mortality in low resource countries. Much of this effort has focused on increasing rates of antenatal care, ensuring that deliveries are attended by skilled birth attendants, and making sure that these deliveries occur in a healthcare facility. After years of hard work and millions of dollars in investment, a growing body of evidence suggests that resource poor areas with increased antenatal care and skilled birth attendants are not seeing the expected proportional drop in maternal mortality [21].

Following the failure to reach the MDGs’ maternal mortality goals, the creation of the SDGs in 2015 included a target for maternal mortality of less than 70 deaths per 100,000 live births [22]. A blueprint for reaching this goal was published in the Lancet Maternal Health Series in 2016. A key step in achieving this goal is improving the quality, and not simply the QoC [23]. In discussing an approach to QoC evaluation, we will focus on a discussion on its application to maternal health, using examples related to this field.

## Methods

Three sources of information informed the development of this paper:

(a) Literature reviews of the QoC interventions and evaluations of complex intervention experience.

(b) Based on the experience of authors. Many of the authors work in the field of evaluating complex interventions and are based in the Evaluation Centre for Complex Health Interventions in Toronto. One of the co-authors has been a leader in evaluation for close to three decades and has experience in conducting evaluations of complex interventions in many international contexts.

(c) The framework and the approaches have been presented in multiple Evaluation Centre webinars with leaders in the field of evaluation in attendance. The recommendations in this paper have been informed by the feedback received in these webinars.

## Quality of care frameworks

### Existing models of QoC and the need for a new framework

We searched the literature for conceptual frameworks related to QoC. While there are many potential frameworks related to maternal health and QoC in general, we chose well-established frameworks that have been previously used to evaluate maternal health interventions for inclusion here.

There are several prominent models for QoC in the literature. Some are simple, and while succinct, are not easily operationalizable, such as the definition offered by the Institute of Medicine, ‘the degree to which health services for individuals and populations increase the likelihood of desired health outcomes and are consistent with current and professional knowledge’ [24,25].

Definitions or models that define quality using individual dimensions or components begin to recognize the complexity and multidimensionality of QoC. The most cited QoC model is the Donabedian model, which divides QoC into *structure* (i.e. the factors that affect the context of care, such as facility, human resources, and training), *process* (i.e. the actions that comprise healthcare, both what is delivered and how) and *outcomes* (i.e. the effects of the healthcare on the individual or population) [26].

Other prominent models include that of Hulton et al. [27], which proposed 10 elements as a framework for maternal QoC and separated these 10 elements into two components: (1) the quality of the provision of care (human and physical resources, the referral system, maternity information systems, use of appropriate technologies, internationally recognized good practice, and management of emergencies) and (2) the quality of the care as experienced by users (human and physical resources, cognition, respect, dignity, equity, and emotional support) [27].

In their review of the existing literature, Raven et al. [28] examined current frameworks used to approach QoC in maternal and newborn health and identified several focus areas. Current models include those based on assessing QoC from the user’s experience of care, the client’s perspective, patients’ rights and providers’ needs, and input–output-outcome models [29].

A framework that assesses QoC must also recognize the interdependence of the various dimensions of quality. This is critical in deciding which levers to push and, therefore, where to invest in order to drive improvements in quality. For example, in India, an intervention that provided cash transfers to women to deliver their babies in healthcare facilities failed to achieve an adequate level of skilled birth attendance. In this case, the primary barriers identified included abusive staff and a lack of person-centered care [30]. The intervention incentivized technical quality (facility delivery, attendance of skilled birth attendant), but failed to recognize a connection between technical quality and person-centered care.

Consistent with a view of complex adaptive systems, QoC is not a measure of a single interaction in the healthcare system but is rather a result of multiple interactions at various levels of care (primary, secondary, tertiary). QoC must also be viewed as dynamic, rather than episodic, in nature, as it extends beyond isolated interactions within the healthcare system and must encompass the supports and resources available outside the healthcare system because these shape a patient’s interaction with the healthcare system. For example, a recommendation of bedrest in treating pre-eclampsia may be unrealistic for a rural woman living in poverty who has no support and little choice but to continue with her daily activity. In a low resource setting, quality varies not just between countries, but also within countries, within states, within cities, and even within individual facilities.

Finally, we argue that a framework for quality must recognize that not only is quality dynamic, but there is a dynamic interdependence between the components of quality. For example, if good quality care is a function of the care given and the care experienced, we know that this contributes to engagement with care over time [31]. We also know that increased engagement leads to better quality care [32]. As the various dimensions of quality improve, a robust framework must provide the user direction to other dimensions that may be affected by this change and may be prudent to measure.

In the next section, we synthesize the above definitions into a novel framework, while also exploring the implications for evaluation of each of the dimensions.

### Towards a multidimensional view of quality of care

**Figure 1.**
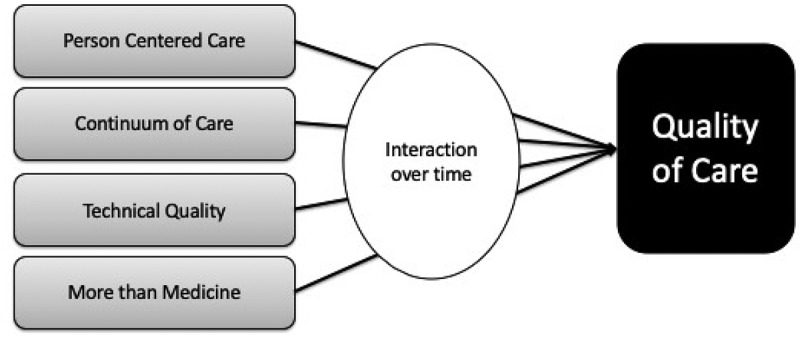
Proposed framework for quality of care

Based on the existing literature and our experience with QoC evaluations in resource poor settings, we propose the following framework consisting of four core domains: (i) person centered care, (ii) continuum of care, (iii) more than medicine, and (iv) technical quality. The framework allows us to map theoretical relationships, create testable propositions, and help inform the development of an evaluation framework (see Figure 1). Our framework has been informed by the House of Care model from the UK [33] that focuses on coordinated care for patients with long-term conditions.

In what follows below, we describe both the conceptual implications and the implications for evaluation for each of these core domains.

### Person-centered care

Person-centered care is the delivery of care that involves patients in shared decision-making with health care providers. It takes into consideration their values and priorities and treats them as an active contributor to their health, rather than a passive recipient of healthcare [34]. When viewing healthcare as more than just a series of interactions with a healthcare provider, it becomes clear that without the participation of the patient, patients are less likely to follow direction, take prescribed medication, or participate in positive activities, such as attending regular checkups or getting immunized [35]. The importance of person-centered care has been shown to lead to improved health outcomes and utilization, particularly in areas of socio-economic, cultural, or religious diversity [36]. Improvements in person-centered care measures show a correlation with ‘increased adherence to modern family planning methods, […] shorter labor, better coping with pain, decreased incidence of operative birth, increased incidence of spontaneous vaginal delivery, increased maternal satisfaction, less anxiety, and increased rates of breastfeeding initiation’ [17].

In their 2017 Person-Centered Care Framework for Reproductive Health Equity, Sudhinaraset and colleagues outline eight patient reported measures around dignity, autonomy, privacy/confidentiality, communication, social support, supportive care, trust, and health facility environment [17]. Monitoring implementation using such a framework and conducting periodic assessments of the person-centered policies and practices among healthcare staff and healthcare facilities might be vital to changing the landscape of clinical practice in regions with poor maternal outcomes.

**Table:** 

Implications for evaluation of patient-centred care
An evaluation approach to person-centered care will help identify the choices and preferences of clients. In systems that are poorly resourced and under capacity, individuals might not feel empowered to express their choices and preferences. In such cases, evaluation may have a role to play in helping create conditions for patients to express their voice regarding their own choices and preferences. This is a hard problem because, in our experience, the shift from patients as recipients of healthcare to being active players in the co-production of their health requires a corresponding shift in values from the multiple actors involved in the delivery of healthcare. Evaluation itself might have a role to play in helping create awareness among healthcare providers of the importance of clients’ voice. Different evaluation methodologies might be needed to better understand patient preferences. One example of a research and evaluation methodology that can be used in this context is brokered dialogue [37], a methodology in which video is used to better understand the nuanced points of view of the different actors involved in health production in a non-confrontational way. Similarly, participatory evaluation approaches [38,39] can be useful in helping understand how interventions can empower clients. Some critical questions that evaluation will need to address in relation to person-centred care include: What are the processes by which women, who have not formerly been regular users of healthcare systems, feel empowered to express their preferences and choices? What kinds of spaces promote dialogue and understanding between healthcare providers and clients? What are ways in which there can be non-confrontational enhancements of dignity and respect toward clients?
Each of these questions must be addressed in a contextually informed way, paying attention to the political and cultural constraints of specific healthcare settings and participants.

### Continuum of care

Health care delivery is frequently siloed. Healthcare providers dealing with one issue often lack a comprehensive picture of a patient’s health and are often unaware of what other providers have planned. A lack of integration across the continuum of care results in, at best, wasted resources and redundant care; at worst, it can result in harm. For example, efforts to ensure high rates of skilled birth attendance may result in little improvement in neonatal and maternal mortality if there exists a gap in connecting newborns to timely and repeated follow-up care within the first weeks of life, when they are most vulnerable.

Maternal and neonatal care must be viewed not as a stand-alone entity, but instead as interwoven in the framework of existing healthcare and women’s homes and community. A continuity or continuum of care must exist throughout a woman’s lifecycle, from adolescence, pregnancy, childbirth, and childhood. As Kerber argues, ‘Saving lives depends on high coverage and quality of integrated service-delivery packages throughout the continuum, with functional linkages between levels of care in the health system and between service-delivery packages, so that the care provided at each time and place contributes to the effectiveness of all the linked packages’ [40].

A care continuum also suggests minimizing programming silos as these can affect health system resilience [41]. Perinatal mortality through indirect causes, such as HIV/AIDS, malaria, and non-communicable diseases are on the rise and comprehensive care packages should be linking existing services for these diseases with maternity services for optimal efficiency and cost-effectiveness [23]. Having such routine, reliable, comprehensive care that follows a person throughout her life-cycle will promote ‘the adoption of healthy behaviors and empower individuals and families to demand quality services’ [40], which, in turn, can perpetuate the use of, and a reliance on, the health system.

Epidemiological and health systems contexts need to form the bedrock of continuum of care measures for maternal and neonatal healthcare. Care fragmentation is prevalent in developing countries simply because ‘human-resource capacity, health-facility infrastructure, supply systems, financial resources, government stewardship, district-level management, and monitoring’ are poorly coordinated [40]. Some basic administrative measures that can strengthen care continuity include linking data through the use of technology, where there is capacity, or training providers to keep accurate medical records [42,43]. It is vital that national and subnational bodies ensure that continuum of care measures not merely test service utilization, but also the quality and level of integration between services [44,45]. This is critical so that women and children are not lost from one medical episode or instance of service utilization to the next.

**Table:** 

Implications for evaluation of continuum of care
In our experience, it is rare that the continuum of care in a setting is clearly mapped. A key role for evaluations is thus to more clearly map the continuum as well as the gaps in such a continuum. One important purpose of evaluations will involve following women longitudinally across their journeys 46 through the continuum of care of the maternal health system. In fulfilling these functions, evaluations should consider some of the following questions What are critical gaps in the continuum of care? Are disadvantaged areas especially deficient in the connectivity across the continuum? What are solutions to enhance the connectivity of especially disadvantaged clients across the continuum?
Evaluations can help both in understanding which types of interventions can assist in navigating the continuum, as well as the spatial [47,48] and network characteristics [49,50] of well-functioning continuums in specific contexts.

### More than medicine

We know that community-based care has the potential to lower maternal mortality [51,52]. Even in the most conventional clinical setting, good anticipatory care needs to be informed by an understanding of the types of supports available to the client. The dimension of ‘more than medicine’ extends beyond structured community resources to include understanding of healthcare recipients’ partner, family, and other interpersonal supports. Currently, in India, the health sector is largely absent from the social milieu of the perinatal period [53], despite good social support being a predictor of lower maternal morbidity [54].

More than medicine in a maternal care setting mobilizes a ‘multipronged attack from all conceivable fronts – using both obstetric and non-obstetric interventions’ [55]. For example, development interventions through primary education, microcredit, and women’s empowerment initiatives have seen a reduction in violence against women, increased family-planning practice, and improved children’s nutritional status [55].

Measures of ‘more than medicine’ could include whether the health care practitioners have promoted community health resources, assessed the needs, goals, and limitations of patients, established an understanding of community resources, and aligned these resources with patient and population needs [56]. Health system stakeholders could also look for the presence of ‘peer support services, advocacy services, structured education, [and] coaching programmes’ [56] targeted at women’s education, empowerment, health, and nutrition education. Mobilization of civil society groups and the voluntary sector could also be markers of robust community support structures, while continued advocacy on behalf of women and mothers would be instrumental in shifting local priorities to addressing challenges unique to women.

**Table:** 

Implications for evaluation of more than medicine
An evaluation of the ‘more than medicine’ dimension will explore the if and how care being delivered in a facility is sensitive to the kinds of support systems that women have at home or in the community. Evaluations can also explore if women whose trajectories of outcomes are more favorable also live in households/communities that have greater supports. From a more developmental perspective, an evaluation could also explore how care in the facility needs to understand and incorporate the types of support systems that exist both within the household and within the community within the care process [57]. Such considerations become especially important if one considers the inequity aspects of quality of care [58,59]. A forward-looking evaluation could play a valuable role in helping healthcare providers identify community-level responses to inequities.

### Technical quality

At its simplest, technical quality refers to safe, accurate, and well-timed routine and emergency clinical interventions done by competent staff who are trained according to internationally recognized and evidence-based guidelines of care delivery. However, an assessment of technical quality must consider more than just the provision of a clinical procedure or the competency of the provider. For example, knowledge of the appropriate use of uterotonic drugs is meaningless without a procurement system to ensure readily available supply of those drugs. Similarly, training in the appropriate administration of antibiotics will have limited impact without the presence of a robust accountability system to ensure the behavior continues beyond the observation period of an intervention. Technical capacities vary dramatically between facilities and across locales based on the resilience of their physical and human resources, adherence to good practice, and the presence of robust accountability systems.

From a clinical standpoint, the WHO recommends eight signal functions for obstetric care that all facilities should meet [60]. Additional comprehensive services would include facilities being able to perform surgeries (e.g. cesarean section) and perform blood transfusions [60]. High rates or mortality and morbidity, despite technical quality improvements, potentially suggest that other dimensions are deficient. Emphasis on measures of efficiency through appropriate levels of funding, the cost-effectiveness of interventions, and effective administration are important in establishing and sustaining quality improvements [61].

**Table:** 

Implications for evaluation of technical quality
While evaluations can help identify whether good quality of care is associated with longer term outcomes, it is helpful to adopt a longitudinal evaluation approach in order to understand if good quality clinical care has differential trajectories of outcomes associated with different types of clients. There is value in assessing potential inequities in impacts in good quality clinical care for different types of clients. Additionally, evaluation can help understand the longitudinal relationship between the different dimensions of quality of care to longer term outcomes. Put differently, what proportion of longer-term outcomes are driven purely by the clinical dimension of care? Does technical quality have differential impacts on differential social groups due to a range of other factors? Even for this technical dimension, there is value in both conducting a summative evaluation, as well as addressing questions that explore the connections between this dimension of quality to other dimensions of quality.

## Discussion

This paper has been informed by an understanding that views of QoC need to be informed by a recognition that quality is a dynamic, emergent aspect of complex adaptive systems [2]. We have argued that evaluations need to promote knowledge of how quality can emerge given the complexities of context, the dependence of interventions on underlying systems, and the necessity for adaptation of interventions to specific contexts. Our primary argument is that we need to enhance our toolbox of evaluation approaches that are currently used to understand and enhance QoC. Participatory evaluation approaches [38,39], for example, can help incorporate patient/client voices as part of better understanding the heterogeneities of person-centered care. A realist evaluation [12,13] approach, on the other hand, can assist in better understanding how interventions need to adapt to specific contexts in order to trigger specific mechanisms that can impact outcomes. The developmental evaluation approach [10,11], moreover, recognizes that interventions being implemented in specific contexts are often incomplete in their knowledge of context. From a developmental evaluation perspective, the evaluation itself plays an important role in helping such interventions adapt to specific contexts.

The paper has identified the following implications for evaluating QoC:

1. *Recognition of the multiple purposes of evaluation*: This paper draws attention to the multiple purposes of evaluation in understanding and enhancing QoC. Returning to the formulation of QoC as an emergent property, we believe that evaluations are most useful if they are situated within a learning system [62,63] that pays attention to improvements over time. Such improvements can be achieved not merely by providing evidence of good QoC, but also by promoting thinking about what constitutes good QoC, developing theories of change to address context-specific impediments to QoC [64–67], and developing an enhanced theoretical understanding of the heterogeneous pathways by which good QoC can impact individuals [68].
2. *Clarifying the role of context in implementing QoC interventions*: In our experience working with complex interventions, it is rare that interventions are clearly fungible across contexts. Given the challenges of explaining variations in QoC across contexts, we argue for a realist evaluation perspective; the focus of realist evaluation is on the contexts and mechanisms that generates the outcomes. Realist evaluation falls into the class of theory-driven evaluation approaches that seeks to explain ‘what works for whom, in what circumstances, in what respects, and how?’ [69]. Table 1 describes some of the key tenets of realist evaluation and why it is particularly suited for iterative learning for QoC. Evaluation, thus, needs to promote thinking about how interventions can be implemented with high ‘fidelity’ across multiple contexts [65,67,70]. In our view, context (and support structures) are often under-theorized in most descriptions of QoC interventions. We suggest that evaluations need to promote a more formal approach to thinking about the role of context and support structures in facilitating or hampering the implementation of interventions. Incorporating context has important implications for how programs are planned and implemented. For example, a good theory of change will need to describe the infrastructure and institutional supports that are necessary for interventions to be successful [71].
3. *Minimal thresholds of QoC*: One of the additional advantages to thinking evaluatively about the multiple dimensions of QoC is the attention such thinking brings to the minimum standards for each of the dimensions. For instance, even though there has been a lot of research focus on the minimum threshold for good clinical care [72], there has not been much research done on the thresholds for person-centered care. Abusive care, for example, can lead to clients dropping out of treatment altogether. A focus on minimal standards of care also can shed light on the capacities of health systems needed to provide high-quality care. For example, severely over-stretched health professionals might not be in a position to provide good levels of person-centered care because of time constraints placed on interactions with patients.
4. *Developing a research agenda for QoC*: Another strength of thinking evaluatively about the multiple dimensions of QoC is that it can lead toward generating hypotheses that can be explored in the future research and evaluation. Some examples of longitudinal evaluation questions that emerge from the framework presented in this paper include:
  - Can more favorable trajectories of person-centered care lead to an enhanced likelihood of the safe delivery of healthy babies?
  - What is the relationship between clinical care and person-centered care in high-quality health systems?
  - Do increased supports at home and in the community lead to a greater likelihood of safe deliveries?

**Table 1.** Relevance of realist evaluation for iterative learning

Tenets of realist evaluation (70)	Implication for building iterative learning for quality of care
‘The intervention is a theory or theories’	There is a need in the literature to specify both direct and indirect pathways by which Quality of Care interventions can impact health outcomes. A number of Quality of Care interventions are undertheorized – the mechanisms by which quality of care interventions impact outcomes are not fully described; a number of Quality of Care interventions lack a well specified theory of change.
‘The intervention involves the actions of people’	In our experience, often the technical aspects of the quality of care interventions are fully described. The role of human relationships and the human capabilities needed to enhance quality and health outcomes are not often clearly described. Further the systems that can help the ‘relationship’ dimensions of Quality of Care are not often clearly specified.
‘The intervention consists of a chain of steps or processes’	Taking a complex adaptive system view towards Quality of Care implies a need to pay attention to the linkages between the multiple dimensions of quality of care. Further, the linkages between enhanced Quality of Care and health outcomes might be dynamic. There is a need to pay attention to the dynamic linkages between the multiple dimensions of Quality of Care as well as the linkages between Quality and client-level health outcomes.
‘These chains of steps or processes are often not linear, and involve negotiation and feedback at each stage.’	The effectiveness of any one component can be enhanced through feedback between the components. From an analytical perspective, while it may make sense to separate out the multiple dimensions of Quality of Care, the actual experience of the different dimensions as experienced by the client may not be as discrete over time.
‘Interventions are embedded in social systems and how they work is shaped by this context.’	The same quality of care intervention might work very differently in different settings. Part of an iterative learning approach will require ‘principled’ adaptations to specific contexts.
‘Interventions are prone to modification as they are implemented.’	An iterative learning strategy needs to inform adaptations in the quality of care interventions over time. These adaptations can be driven by the heterogenous needs of clients that might surface over time or as a response to the 5Is of contexts (infrastructural, institutional, interpersonal, individual and intersectional components of contexts)
‘Interventions are open systems and change through learning as stakeholders come to understand them.’	There is a need for a learning system that promotes dialogue and learning between stakeholders. Without such an intentional learning system, opportunities to improve system and intervention-levels of Quality of Care can be lost.

### Towards principles of evaluating quality of care

Following the core principles enunciated by the LGHC, this paper argues that evaluations of QoC initiatives also need to be informed by some critical principles that move beyond a clinical view of QoC. Following the QoC framework presented in this paper, we argue that evaluations of QoC interventions should be guided by the following principles:

1. Pay attention to clients’ journeys: Our conceptualization of quality recognizes that measuring the quality of a single episode of care needs to be embedded within the client’s journey. This follows from a recognition in LGHC that health systems are ‘for people.” It is important to recognize that the client herself is a co-producer of her/his own health outcomes [73]. This, of course, means asking important questions of the intervention, such as whether the intervention empowers the client as a co-producer of her own health [73] and whether a client feels that the intervention gives her a voice to state her preferences and choices [73].
2. Respect and dignity matter: As part of any multi-dimensional measurement of QoC, we think that there needs to be a greater focus on the principles of respect and dignity [74–76]. It is important for an evaluation to explore if an intervention treated a woman with dignity and respect. It is likely that respectful care can be a means to better long-term outcomes.
3. High-quality care is anticipatory: Our conceptualization is also driven by a recognition that high-quality care needs to anticipate key events in a client’s life, and also anticipate constraints and barriers. Evaluations of QoC also need to interrogate whether care is anticipatory [77]. High-quality care is driven by a view of key long-term outcomes that need to be achieved – for example, a safe birth. We need to think longitudinally in terms of the trajectory of care, as it is a journey made up of multiple key events over a period of time. In the context of maternal health, the central focus thus needs to be on what is needed to ensure a safe delivery and a healthy baby and mother over time.
4. High QoC pays attention to contexts: There are multiple types of contexts that might influence whether a client received good quality care. Such care can be grouped into what we characterize as the *5I’s* – which refers to Infrastructural, Institutional, Interpersonal, Individual, and Intersectional considerations [78–80]. In our experience, variations in the 5I’s can lead to considerable heterogeneities [81,82] in the QoC women experience. Given the heterogeneities that are introduced by the realities of the 5I’s, providing quality care is rarely a mechanical process. For example, when a health care professional is faced with a high-risk pregnancy, attention needs to be given to determine the types of care that client can receive in her home, the types of supports she has available, the type of preferred interpersonal communication that will facilitate adherence to treatment, the types of work obligations she might have, and the types of transportation she has access to in order to get to a district-level facility that can deliver high-quality care for potential complications. Evaluations of QoC initiatives need to pay attention to the 5I’s and explore the role of multiple contexts in leading to variations in QoC.
5. Quality of care is system-aware

All health care providers are embedded in larger systems [83–87]. Delivering high quality care is, therefore, dependent on many systemic factors including having a well-functioning human resources system, a procurement system that pays attention to shortages of key medicines, and so on. Ideally, an evaluation of QoC initiatives will be able to provide feedback on role of system-level factors in enhancing the efficacy of QoC interventions.

### Towards system-level learning

Taking a systems perspective to QoC, LGHC (1) has argued that ‘… evidence from health and other sectors shows that complex adaptive systems can thrive if actors within the system have a shared vision, clear rules, and space to allow evolution and learning.’ An important question for evaluation is: How can a learning system promote such evolution and learning? Table 2 describes some questions that comprehensive evaluations that are both context and capacity-aware and guided by system-level learning need to explore. We recognize that realistically a number of the questions are not relevant to most QoC interventions; yet, we think such a list of questions will help the planning and knowledge translation of QoC interventions. Addressing some of the questions can also enhance the utility and relevance of evaluations.

**Table 2.** Questions to guide a comprehensive approach to QoC evaluations

Evaluation focus	Questions for reflection
Purpose of evaluation	Does the QoC intervention need further development? If yes, how can evaluations help? Is the intervention ‘mature’ for a summative evaluation? If not, what are areas that need further development? If yes, what kinds of impacts will be explored in the evaluation? Is there a need for a mixed evaluation approach with an initial developmental approach and then movement to a summative evaluation?
Information and learning needs	Who are the key stakeholders? What do they need to learn? Understand the types of information that evaluation can provide that can help with implementation and learning
Theory of change	Does the intervention have multiple components (focussing on multiple dimensions) or is the focus mainly on just one dimension? Are the linkages between the different components clearly described? Is there ‘coherence’ in the overall causal package? Is there a clear theory of change of the intervention? Are the mechanisms by which QoC interventions can impact health outcomes clearly specified? Is there clarity about the types of contexts/support structures that will facilitate the linkages between QoC dimensions and health outcomes?
Context mapping	Is there clarity on the contexts that either support or hinder the implementation of the QoC interventions? How will the intervention need to be adapted to the contexts? What infrastructural, institutional, interpersonal, individual and intersectional contexts does the program need to respond to?
Capacity mapping	Are the human, technical and administrative capacities of the program sufficient to implement the multiple interventions? Can the evaluations help identify in which dimensions are the capacities insufficient? Can key capacities be enhanced by actions from other actors in the system?
Anticipated impacts	How long will it take for the QoC intervention to improve health outcomes? Are there ideas on the anticipated trajectory/shape of the impacts? Will the proposed timeframe of the evaluation match the anticipated timeline of impacts?
Implementation fidelity and adaptation	Are sufficient adaptations being made to adapt to contexts? Is implementation integrity being maintained over time? Does the evaluation provide any information to help with adaptations?
Impacts	What evaluation designs are being implemented to study the impact over time of the QoC interventions on health outcomes? Are the impacts stable over time? Does the intervention impact different social groups differentially? Is there evidence of health equity impacts? Does the evaluation help develop clarity on the key drivers of impacts? Does the evaluation shed light on the contexts necessary for maximal impacts? Does the evaluation shed light on ‘what works for whom’?
Planning for sustainability	Are there explicit plans to sustain the intervention? How can the evaluation help with making decisions to sustain the intervention? Can the intervention be institutionalized? Can key learnings about effective mechanisms be mainstreamed?
Scaling-up	Is there a need in the overall system to implement such an intervention? Do the impact results support spreading of the intervention widely? Does the overall system have the capacity to implement this intervention? Can the mechanisms be spread across the system?
System-level learning and feedback	What can be done to improve each component of the QoC intervention? How can the linkages between the multiple QoC components be strengthened? How can the linkages between organizations in the system be strengthened? Are some key organizations missing from the delivery network? Can the linkages with community organizations be strengthened? What is a learning and communication system that can promote a learning culture around person-centered care?

### Limitations and potential strengths

We recognize that this expanded view of evaluation will carry with it some limitations and potential objections. One concern we anticipate to the multiple purposes of evaluation described in this paper is that it might inadvertently be making evaluation harder than it needs to be by focusing on data in multiple dimensions. While we recognize that taking the multiple dimensions of QoC seriously does imply an additional data burden, we think the ‘learning pay-offs’ associated with such additional data will more than make up for any burdens created by such a focus on multiple dimensions of QoC. This paper was a response to how the evaluation community can be responsive to the recent LGHC on high-quality health systems. The iterative view recommended by the LGHC would require, in our view, a much more diverse and pluralistic approach to evaluation and data collection. We also think that taking such a pluralistic view of evaluation itself also will help move the field away from a mechanical view of evaluation in which products often are developed without serious learning occurring. Additionally, the view espoused in this paper, driven by our experiences working in developing country settings, is that interventions are not often transportable across contexts. The adaptations required for interventions to be ‘adapted’ into different contexts does imply the need to think evaluatively on how best can interventions be implemented across contexts.

We recognize implementing the conceptual ideas discussed in this paper will remain challenging. Operationalizing many of the ideas, such as information systems for ‘more than medicine’ or discuss recent innovations in data linkages, or more recent advances in analytical methods will require another paper with a narrower focus on a specific intervention. We acknowledge the absence of discussion on measurement, data collection, data linkages, and analysis as a limitation of this paper.

## Conclusion

We have argued that an expanded view of evaluation can help shift current definitions of QoC as a primarily clinical phenomenon toward a multidimensional view that prioritizes the respect for and dignity of the client. We also suggest that it is vital that a view of quality does not treat the individual as a passive player in the production of good health outcomes. Instead, a view of quality needs to pay attention to the critical role played by the client in the co-production of health. Thoughtful evaluations can play an important role in helping to move toward an expanded conceptualization of quality. This paper has relevance for the role of evaluation in helping meet the SDG goals of maternal health. High-quality health systems will be critical to meeting the SDG targets. There is a need for a discourse around the multiple roles that evaluation can play in enhancing the likelihood that the SDGs focus on ‘no one left behind’ can be successful.

## Paper context

This paper explores the role of evaluation in building iterative learning to achieve the Lancet Global Health Commission’s recommendations for achieving high-quality health systems through iterative learning. The multiple role evaluation can play in achieving such iterative learning is described by presenting a novel quality of care model that was adapted to maternal health settings. Key dimensions of the model include person-centered care, clinical care, continuum of care, and ‘more than medicine.’
